# Caste-specific expression of genetic variation in the size of antibiotic-producing glands of leaf-cutting ants

**DOI:** 10.1098/rspb.2009.1415

**Published:** 2009-10-28

**Authors:** W. O. H. Hughes, A. N. M. Bot, J. J. Boomsma

**Affiliations:** 1 Department of Biology, Centre for Social Evolution, University of Copenhagen, Universitetsparken 15, Copenhagen 2100, Denmark; 2 Institute of Integrative and Comparative Biology, University of Leeds, Leeds LS2 9JT, UK; 3 Department of Ecology and Genetics, Institute of Biological Sciences, University of Aarhus, 8000 Aarhus-C, Denmark

**Keywords:** phenotypic plasticity, castes, heritability, parasite resistance, genetic diversity

## Abstract

Social insect castes represent some of the most spectacular examples of phenotypic plasticity, with each caste being associated with different environmental conditions during their life. Here we examine the level of genetic variation in different castes of two polyandrous species of *Acromyrmex* leaf-cutting ant for the antibiotic-producing metapleural gland, which has a major role in defence against parasites. Gland size increases allometrically. The small workers that play the main role in disease defence have relatively large glands compared with larger workers, while the glands of gynes are substantially larger than those of any workers, for their body size. The gland size of large workers varies significantly between patrilines in both *Acromyrmex echinatior* and *Acromyrmex octospinosus*. We also examined small workers and gynes in *A. echinatior*, again finding genetic variation in gland size in these castes. There were significant positive relationships between the gland sizes of patrilines in the different castes, indicating that the genetic mechanism underpinning the patriline variation has remained similar across phenotypes. The level of expressed genetic variation decreased from small workers to large workers to gynes. This is consistent with the hypothesis that there is individual selection on disease defence in founding queens and colony-level selection on disease defence in the worker castes.

## 1. Introduction

The expression of disease resistance mechanisms is highly variable and dynamic, but the selection factors that maintain this variation in space and time are still poorly understood. Disease resistance mechanisms are generally costly, often substantially so, and investment in disease resistance is thus traded off against other traits that may be essential to the reproductive success of an individual (Kraaijeveld & Godfray 1997; Poulsen *et al*. 2002*b*; Rolff & Siva-Jothy 2003; Vijendravarma *et al*. 2009). In addition, the parasite community that an individual will encounter during its life will be diverse and rarely predictable. Different parasites may well require different resistance mechanisms and, as parasites will vary in virulence, there will be different optimum levels of investment in resistance for different parasites. As a result, genetic variation in disease resistance is ubiquitous (Ebert 1998; Little & Ebert 1999, 2000, 2001; Carius *et al*. 2001; Kover & Schaal 2002; Baer & Schmid-Hempel 2003; Hughes & Boomsma 2004).

In many organisms, variation in disease resistance is further increased by phenotypic plasticity, in which a single genotype may express multiple phenotypes according to environmental conditions (Pigliucci 2005). The interaction with parasites, and thus the benefits of investment in a particular defence mechanism, will differ between phenotypes. For example, both locusts and armyworms can develop into either gregarious or solitary phenotypes. Parasite pressure is greater for the former than the latter, and accordingly so is the investment in disease resistance (Reeson *et al*. 1998; Wilson *et al*. 2002). The potential interaction between genetic and phenotypic diversity also needs to be considered. In phenotypes in which resistance and fitness are strongly linked, for example due to common infection by a highly virulent parasite, the level of expressed variation is likely to be restricted. In phenotypes where the link between resistance and fitness is weaker, the level of expressed genetic variation may be greater as a wider range of resistance levels may be successful according to the trade-off with other traits. The level of expressed genetic variation for disease resistance can therefore be expected to differ between phenotypes.

Social insects include some taxa, such as leaf-cutting ants (*Acromyrmex* and *Atta*), that are outstanding examples of both genotypic and phenotypic diversity. Unlike most social insects, which are monandrous (Hughes *et al*. 2008*a*), leaf-cutting ant queens mate with multiple males and produce genotypically diverse offspring (workers and queens) in monogynous colonies (Villesen *et al*. 2002; Ortius-Lechner *et al*. 2003; Sumner *et al*. 2004). This facilitates the detection of genotypic effects because same-cohort offspring of different fathers (patrilines) within a colony share the same maternal genotype on average, the same maternal effects, and the same environmental rearing conditions, so differ only in their paternal genotype. In addition, leaf-cutting ants have both morphologically distinct queens and workers, and highly polymorphic workers (Wilson 1980; Wetterer 1999; Hughes *et al*. 2003). Queens gain their fitness through direct reproduction and are thus under individual-level selection, whereas colony-level selection is most important for workers as they gain their fitness indirectly through the reproduction of their kin. Furthermore, although the within-colony environment of the different worker castes may be identical, each of them expresses adaptations in response to different selection pressures. Some, such as *Acromyrmex* large workers, forage outside of the nest or work with hazardous waste, while others, such as *Acromyrmex* small workers, spend their entire lives as nurses and fungus gardeners within the nest (Wetterer 1999; Hughes *et al*. 2003). Queens are faced with very high disease pressure when founding colonies (which they do independently), but are protected by workers before and after this stage. The environmental conditions experienced over the lifetime of the different castes can therefore be markedly different and they may express different disease resistance as a result.

A key component of disease defence in leaf-cutting ants is the metapleural gland. This paired structure consists of bundles of secretory cells, a collection sac and a storage chamber (the bulla), which forms a visible bulge in the exterior surface (Hölldobler & Wilson 1990; Bot & Boomsma 1996; Bot *et al*. 2001). The secretion from the gland contains at least 20 compounds (Ortius-Lechner *et al*. 2000*b*), many of which have antibiotic properties (Bot *et al*. 2002). Ants actively spread the secretion over their cuticle and mouthparts, and also actively apply it to the fungus garden, reducing the viability of parasite spores (Hughes *et al*. 2002; Fernandez-Marin *et al*. 2006, 2009). The secretion makes ants with functional glands substantially more resistant to parasites (Poulsen *et al*. 2002*b*) and is also likely to be important in protecting the mutualistic fungus garden against competitive or pathogenic micro-organisms. The former, individual-level effect will be particularly important for queens during the vulnerable colony-founding stage, while the latter role in colony-level defence may be most significant for the small workers thought to play the major role in protecting the colony against parasites (Jaccoud *et al*. 1999; Hughes *et al*. 2002; Poulsen *et al*. 2002*a*, 2006). Metapleural gland function is also costly, consuming some 13–20 per cent of a worker's basal metabolic rate (Poulsen *et al*. 2002*b*), making it particularly likely to be traded off against other traits. Indeed, there is comparative evidence for such a trade-off, with the leaf-cutting ants having evolved substantially larger metapleural glands than all the other members of their tribe owing to a change in parasite pressure (Hughes *et al*. 2008*b*) and a socially parasitic species having evolved smaller glands because it relies on its host for protection (Sumner *et al*. 2003). Investment in the metapleural gland by leaf-cutting ants thus seems likely to vary between caste phenotypes, both in the overall mean and in terms of expressed genetic variation for the trait. Specifically, we would predict low heritability for metapleural gland size in gynes, consistent with Fisher's fundamental theorem (Fisher 1930), but higher heritability in the worker castes because their individual disease resistance is not linked to direct fitness and thus variation may not be removed by selection. Here we test whether these predictions are correct.

## 2. Material and methods

Workers and gynes were sampled from five mature colonies of *Acromyrmex echinatior* (Ae48, Ae125, Ae129, Ae153 and Ae158) and three of *Acromyrmex octospinosus* (Ao67, Ao71 and Ao77) that had been collected from Gamboa, Panama, in 1996, 1999 and 2000. Mature leaf-cutting ant colonies normally produce reproductive individuals in a single bout in the month before the annual mating flight. At the times of collection, all the colonies used were in this phase. For three of the colonies (Ae125, Ae129 and Ae158), workers and gynes were collected together with the main fungus garden and were placed immediately in 96 per cent alcohol. The other colonies were maintained in the laboratory and samples of workers and gynes were later collected from the fungus gardens of each colony on single days. Cuticular coloration provides an estimate of an individual's age (S. A. O. Armitage and J. J. Boomsma 2008, unpublished data), and only individuals of similar coloration were used in order to minimize variation in rearing conditions across nest-mates.

We sampled 50 large workers from each *A. octospinosus* colony, whereas from each *A. echinatior* colony we sampled 94 workers (half large workers and half small workers) and 94 gynes. Individuals were genotyped as described below and assigned to patrilines. The five most abundant patrilines were selected for each colony of *A. octospinosus*, while two to four patrilines that were best represented across all three of the castes were selected for each colony of *A. echinatior*. The metapleural gland was measured for all the individuals sampled from these selected patrilines. This was done directly for *A. octospinosus* using an eyepiece graticule. For *A. echinatior*, a photograph of the metapleural gland of each individual was taken using an Olympus DP-10 camera connected to an Olympus SZX9 binocular microscope and measurements made using the program DP-Soft 3.0. The diameter of the metapleural gland bulla was measured because this has been shown to be a good predictor of the number of gland cells (Bot *et al*. 2001). In order to allow the measurements of metapleural gland diameter to be standardized for variation in body size, a photograph and measurement were also made of the width of the ventral side of the pronotum.

### (a) Molecular analysis

To determine the genotypes of the ants sampled, a single leg (for gynes), a pair (for large workers) or all six legs (for small workers) were removed from the ants. DNA was extracted from the legs using Chelex beads (BioRad; *A. echinatior*) or the CTAB procedure (*A. octospinosus*). The *A. octospinosus* samples were amplified at the microsatellite loci Ech1390, Ech3385 and Ech4126, while the *A. echinatior* samples were analysed as these plus Ech4225 (Ortius-Lechner *et al*. 2000*a*). All microsatellite loci are highly polymorphic in both species so the non-detection error of patrilines was negligible (Boomsma & Ratnieks 1996). Reaction mixes and amplification program were as in Hughes & Boomsma (2006). The *A. octospinosus* samples were amplified in 6 µl volumes of 1 µl DNA, 500 mM KCl, 15 mM MgCl_2_, 100 mM Tris–HCl (pH 9.0), 0.2 mM dNTPs, 0.1 U of Taq polymerase and 2 pmol of each primer. Separate programmes were used for each locus, all beginning with 94°C for 3 min, followed by 39 cycles of 30 s at 93°C, 30 s at the annealing temperature (53°C for Ech1390 and Ech3385l 59.5°C for Ech4126) and 40 s at 72°C, ending with 7 min at 72°C and cooling to 10°C. Hybaid PCR Express and Peltier Thermal Cyclers were used for the amplifications. The products of the polymerase chain reactions were run on 5 per cent polyacrylamide gels with an ALF Express or ABI377 (Applied BioSystems) sequencer and allele sizes were scored by comparison with internal size markers. The multi-locus offspring genotypes were used to infer the genotypes of colony queens and their multiple mates. It was then possible to assign the sampled individuals to patrilines within their colony (six to nine and three to eight patrilines per colony in *A. octospinosus* and *A. echinatior*, respectively). The small number of individuals (approx. 5%) whose paternities could not be established, owing to failed PCR amplification or the individual being heterozygous and having the same alleles as a heterozygous queen at one or more diagnostic loci, were excluded from the analysis.

### (b) Statistical analysis

Both bulla width and pronotum width were log transformed prior to analysis in order to homogenize variances. The bulla widths were then analysed separately for each caste using analyses of covariance in SPSS 14.0 with pronotum width as the covariate, colony as a random factor and patrilines nested within colony. The mean heritabilities (*h*^2^) of relative bulla width were calculated for each caste by dividing the proportion of the total variance explained at the nested patriline level by 0.5. To determine whether any genetic influence on relative metapleural gland size was consistent across the castes, the standardized residuals of the relationship between bulla width and pronotum width were calculated for each caste. We then examined whether the mean residuals of each patriline were correlated for the three castes.

## 3. Results

**Figure 1.**
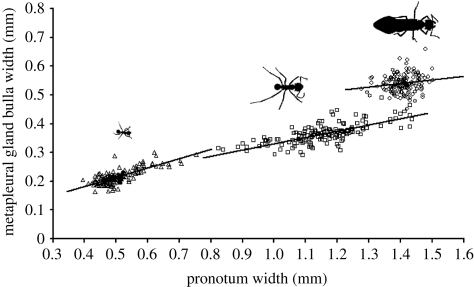
The relationship between metapleural gland size, measured by bulla width, and body size, measured by pronotum width, for small workers (triangles), large workers (squares) and gynes (circles) of *Acromyrmex echinatior*.

The relationship between metapleural gland size and body size differed between the three female castes of *A. echinatior*, increasing relatively steeply in the small workers (*F*_1,136_ = 168.3, *p* < 0.001) and more gradually in the large workers (*F*_1,137_ = 77.1, *p* < 0.001; figure 1). Queens had strikingly larger metapleural glands relative to their body size than did workers and the relationship between the two morphological variables was much less distinct (*F*_1,138_ = 3.42, *p* = 0.067; figure 1).

**Figure 2.**
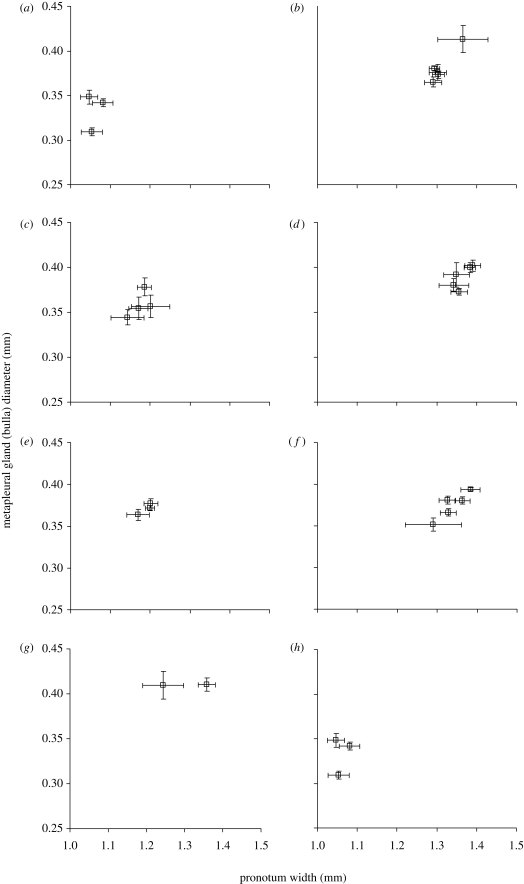
The mean ± s.e. metapleural gland size (measured by bulla width) and body size (measured by pronotum width) of large workers from different patrilines in (*a*,*c*,*e*,*g*,*h*) five colonies of *A. echinatior* and (*b*,*d*,*f*) three colonies of *A. octospinosus*. (*a*) Ae48, (*b*) Ao67, (*c*) Ae125, (*d*) Ao71, (*e*) Ae129, (*f*) Ao77, (*g*) Ae153, and (*h*) Ae158.

The three *A. octospinosus* colonies did not differ in the relative metapleural gland sizes of their large workers (*F*_2,12_ = 0.05; *p* = 0.953), while the five colonies of *A. echinatior* also did not differ in the relative metapleural gland sizes of any of the castes (small workers: *F*_4,10_ = 0.917, *p* = 0.934; large workers: *F*_4,10_ = 1.91, *p* = 0.179; gynes: *F*_4,10_ = 1.49, *p* = 0.272). However, within colonies, the patrilines differed significantly in their relative bulla widths for large workers of *A. octospinosus* (*F*_12,112_ = 3.11, *p* < 0.001; figure 2), and for all three castes of *A. echinatior* (small workers: *F*_10,136_ = 7.49, *p* < 0.001; large workers: *F*_10,137_ = 4.93, *p* < 0.001; gynes: *F*_10,138_ = 2.3, *p* = 0.016; figure 2). Within colonies, patriline explained 30 per cent of the variation in bulla width of *A. octospinosus* large workers, and 43, 27.6 and 12.6 per cent of the variation of *A. echinatior* small workers, large workers and gynes, respectively. The corresponding heritability estimates were 0.608 for *A. octospinosus*, and 0.86, 0.55 and 0.25, respectively, for *A. echinatior*.

Colonies of *A. octospinosus* differed significantly in the pronotum width of large workers (*F*_2,27_ = 4.3, *p* = 0.024; figure 2), while colonies of *A. echinatior* differed significantly in the pronotum widths of their small workers (*F*_4,10_ = 4.19, *p* = 0.026) and large workers (*F*_4,10_ = 12.7, *p* < 0.001) but not their gynes (*F*_4,10_ = 1.11, *p* = 0.401). Within colonies, patrilines did not differ significantly in pronotum width for any of the castes (*A. octospinosus* large workers: *F*_13,128_ = 0.92, *p* = 0.539; *A. echinatior* small workers: *F*_10,137_ = 1.46, *p* = 0.16; large workers: *F*_10,138_ = 1.52, *p* = 0.138; gynes: *F*_10,139_ = 1.64, *p* = 0.1), indicating that genetic variation does not affect overall body size within castes.

**Figure 3.**
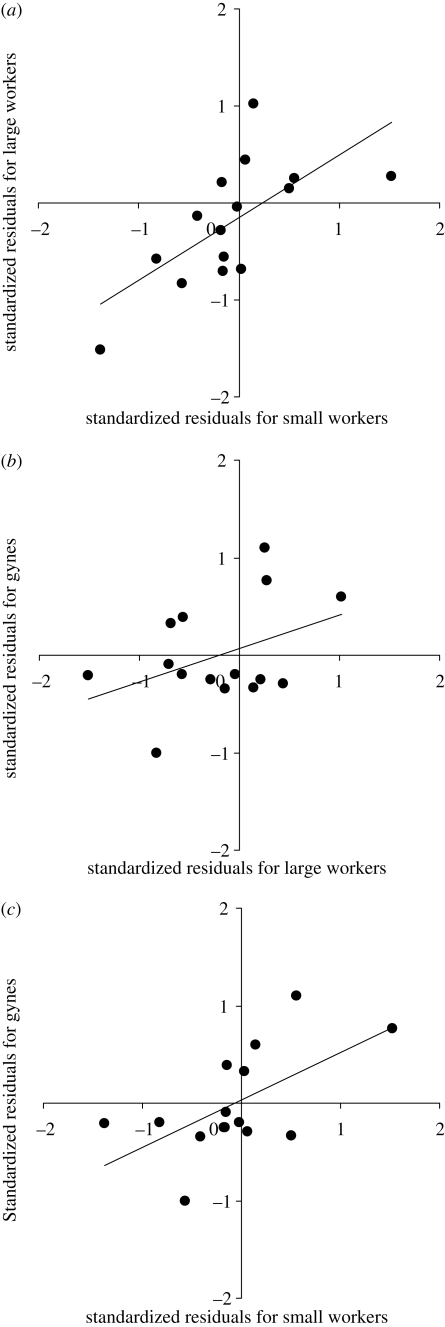
The relationship across patrilines of *Acromyrmex echinatior* between the relative metapleural gland size of the three castes. Standardized residuals of the relationships between bulla width and pronotum width for each caste are used to estimate metapleural gland size relative to body size. The mean (±s.e.) standardized residuals of each patriline are shown for (*a*) small workers against large workers, (*b*) large workers against gynes, and (*c*) small workers against gynes.

The standardized residuals of the relationships between bulla width and pronotum width in *A. echinatior* were significantly positive for the two worker castes (*r*_1,15_ = 0.67, *p* = 0.006) and for small workers against gynes (*r*_1,15_ = 0.595, *p* = 0.019; figure 3). The relationship was also positive for large workers against gynes but not significantly so (*r*_1,15_ = 0.403, *p* = 0.137; figure 3). Patrilines for which small workers had large (or small) metapleural glands relative to their body size therefore also tended to have large workers and gynes with relatively large (or small) metapleural glands.

## 4. Discussion

The high metabolic cost of metapleural gland function, combined with individual-level selection applying to gynes and colony-level selection to workers, led us to hypothesize that there would be both genotypic and phenotypic variation in gland size, and that heritability of gland size would be low in gynes and higher in workers. The results support these predictions. Differences between phenotypes in their investment in disease resistance have previously been described in other animals, such as army worms and locusts, and linked to parasite pressure (Reeson *et al*. 1998; Wilson *et al*. 2002). We found that the size of the metapleural gland relative to body size differed distinctly between all three of the female phenotypes (castes) of *A. echinatior*. As found previously (Wilson 1980; Bot & Boomsma 1996), the slope of the relationship between metapleural gland size and body size was steeper for small than for large *A. echinatior* workers, indicating that the glands of the former were disproportionately large compared with those of the latter. This has been suggested to be because small workers play the key role in preventing unwanted micro-organisms from becoming established in the colony (Kermarrec *et al.* 1986; Jaccoud *et al*. 1999; Hughes *et al*. 2002; Poulsen *et al*. 2002*a*, 2006). Interestingly, though, the relationship for gynes neither followed on isometrically from large workers nor increased allometrically in the manner of the small workers. Rather, the glands were distinctly larger relative to body size for gynes compared to the workers. Given that the glands of large workers consumes some 13–20 per cent of their basal metabolic rate (Poulsen *et al*. 2002*b*), the larger glands of gynes must therefore consume a substantial amount of energy. This makes their size particularly remarkable when put in the perspective of a gyne having to take part in an energetically expensive mating flight followed by a period during which she has to survive largely on her own fat reserves (Weber 1972). Although queens are protected against parasites while in their natal colony, and also once they have produced workers in their own colony, they are highly vulnerable during the intermediate colony-founding stage. It can take 12 weeks before the first adult workers are produced, and the mortality of queens during this period is extremely high, with a substantial portion of the mortality being due to disease (Weber 1972; Baer *et al*. 2006). The relative size of the metapleural glands of gynes indicates that the threat of disease faced by gynes during this period must indeed be consistently high.

In all three castes of *A. echinatior*, as well as the large workers examined for *A. octospinosus*, there was a significant genetic influence on relative metapleural gland size. Such a genetic influence may, at least in part, explain genotypic differences in parasite resistance (Hughes & Boomsma 2004, 2006). Genetic variation in resistance is well known in other animals (Ebert 1998; Little & Ebert 1999, 2000, 2001; Carius *et al*. 2001; Kover & Schaal 2002; Baer & Schmid-Hempel 2003; Hughes & Boomsma 2004), but, within the social insects, a mechanistic basis has only previously been demonstrated in honeybees and bumble-bees (Spivak & Reuter 2001; Baer & Schmid-Hempel 2003; Decanini *et al*. 2007; Navajas *et al*. 2008). The genetic variation in leaf-cutting ant metapleural gland size is most probably maintained because investment in the energetically costly metapleural glands will be traded off against other traits. There is therefore unlikely to be a single optimum metapleural gland size, but rather a range of optima that will depend on the spectrum of parasite pressure experienced by the colony during its lifetime. The positive relationships across patrilines between gland size in the different caste phenotypes suggest that a similar genetic mechanism is involved in each case.

There was no significant genetic influence on worker size within castes. Colony-level selection favours morphological castes because of the benefits of division of labour (Oster & Wilson 1978). Although genetic influences on caste determination occur due to variable optima in caste ratios or selfish cheating (Hughes *et al*. 2003; Hughes & Boomsma 2007, 2008), these results suggest that most genetic variation for size within castes does not become expressed because there are single size optima and thus low heritability. This contrasts markedly with the heritability of metapleural gland size and suggests that while optimum size within a caste is predictable, the threat of parasites is much less so.

Although the genetic influence on relative metapleural gland size was present in all three castes, the heritability of the trait differed. The heritability estimate found for large workers of *A. echinatior* is similar to that found for this caste in the sister species, *A. octospinosus*. Within *A. echinatior*, however, it was greater for small workers and smaller for gynes. It seems likely that these differences reflect the different selection pressures on the three castes. Although small workers may play the key role in preventing micro-organisms from becoming established in the colony's fungus gardens (Jaccoud *et al*. 1999; Hughes *et al*. 2002; Poulsen *et al*. 2002*a*, 2006), they also generally spend their entire life working within the nest, so that their antibiotic defence consists not just of their own secretion but also that of the other small workers with which they interact. In this caste, the metapleural gland defence is therefore largely a collective trait, so that colony-level selection may favour the expression of a wide continuous range of phenotypes around the caste-specific mean values with the expression of patriline-specific genetic variation for metapleural gland size being unconstrained. This contrasts with the body size of workers where caste-specific optima apply and genetic variation around these means is hardly expressed. Gynes, in contrast, play no role in defending the colony against disease and the only function of their metapleural glands will be to defend themselves during the vulnerable colony-founding stage. The range of optimum investment levels in the metapleural gland therefore seems likely to be much more restricted for this caste. Large workers would then appear to be intermediate. They are less important than small workers in defending the colony as a whole against disease (Jaccoud *et al*. 1999; Hughes *et al*. 2002; Poulsen *et al*. 2002*a*, 2006) but, unlike small workers, a substantial portion of their work consists of foraging outside of the colony (Wetterer 1999; Hughes *et al*. 2003). The metapleural gland may therefore have a greater role in this worker caste as an individual defence mechanism than in small workers, but less than in gynes. The seemingly strange pattern of the two female castes for whom the metapleural gland is probably most important having opposite levels of genetic variation may therefore be explained by selection acting on gynes at the individual level and on small workers at the colony level. Although different levels of selection do not apply to the same extent in non-social animals, the trade-off between investment in costly resistance mechanisms and other traits means that individual-level selection for resistance will frequently vary between phenotypes. Differences between phenotypes in the levels of expressed genetic variation may therefore not be as extreme as in leaf-cutting ants, but could nevertheless be significant.

## References

[RSPB20091415C1] Baer B. Schmid-Hempel P. 2003 Bumblebee workers from different sire groups vary in susceptibility to parasite infection. Ecology Letters 6, 106–110 (doi:10.1046/j.1461-0248.2003.00411.x)

[RSPB20091415C2] Baer B. Armitage S. A. O. Boomsma J. J. 2006 Sperm storage induces an immunity cost in ants. Nature 441, 872–875 (doi:10.1038/nature04698) 16778889 10.1038/nature04698

[RSPB20091415C3] Boomsma J. J. Ratnieks F. L. W. 1996 Paternity in eusocial Hymenoptera. Phil. Trans. R. Soc. Lond. B 351, 947–975 (doi:10.1098/rstb.1996.0087)

[RSPB20091415C4] Bot A. N. M. Boomsma J. J. 1996 Variable metapleural gland size-allometries in *Acromyrmex* leafcutter ants (Hymenoptera: Formicidae). J. Kansas Entomol. Soc. 69, 375–383

[RSPB20091415C5] Bot A. N. M. Obermayer M. L. Holldobler B. Boomsma J. J. 2001 Functional morphology of the metapleural gland in the leaf-cutting ant *Acromyrmex octospinosus*. Insectes Soc. 48, 63–66 (doi:10.1007/PL00001747)

[RSPB20091415C6] Bot A. N. M. Ortius-Lechner D. Finster K. Maile R. Boomsma J. J. 2002 Variable sensitivity of fungi and bacteria to compounds produced by the metapleural glands of leaf-cutting ants. Insectes Soc. 49, 363–370 (doi:10.1007/PL00012660)

[RSPB20091415C7] Carius H. J. Little T. J. Ebert D. 2001 Genetic variation in a host–parasite association: potential for coevolution and frequency-dependent selection. Evolution 55, 1136–1145 11475049 10.1111/j.0014-3820.2001.tb00633.x

[RSPB20091415C8] Decanini L. I. Collins A. M. Evans J. D. 2007 Variation and heritability in immune gene expression by diseased honeybees. J. Hered. 98, 195–201 (doi:10.1093/jhered/esm008) 17404328 10.1093/jhered/esm008

[RSPB20091415C9] Ebert D. 1998 Evolution—experimental evolution of parasites. Science 282, 1432–1435 (doi:10.1126/science.282.5393.1432) 9822369 10.1126/science.282.5393.1432

[RSPB20091415C10] Fernandez-Marin H. Zimmerman J. Rehner S. Wcislo W. 2006 Active use of the metapleural glands by ants in controlling fungal infection. Proc. R. Soc. B 273, 1689–1695 (doi:10.1098/rspb.2006.3492) 10.1098/rspb.2006.3492 PMC1634922 16769642

[RSPB20091415C11] Fernandez-Marin H. Zimmerman J. K. Nash D. R. Boomsma J. J. Wcislo W. T. 2009 Reduced biological control and enhanced chemical pest management in the evolution of fungus farming in ants. Proc. R. Soc. B 276, 2263–2269 (doi:10.1098/rspb.2009.0184) 10.1098/rspb.2009.0184 PMC2677613 19324734

[RSPB20091415C12] Fisher R. A. 1930 The genetical theory of natural selection Oxford, UK: Clarendon Press

[RSPB20091415C13] Hölldobler B. Wilson E. O. 1990 The ants Cambridge, MA: Belknap Press

[RSPB20091415C14] Hughes W. O. H. Boomsma J. J. 2004 Genetic diversity and disease resistance in leaf-cutting ant societies. Evolution 58, 1251–1260 15266974 10.1554/03-546

[RSPB20091415C15] Hughes W. O. H. Boomsma J. J. 2006 Does genetic diversity hinder parasite evolution in social insect colonies? J. Evol. Biol. 19, 132–143 (doi:10.1111/j.1420-9101.2005.00979.x) 16405585 10.1111/j.1420-9101.2005.00979.x

[RSPB20091415C16] Hughes W. O. H. Boomsma J. J. 2007 Genetic polymorphism in leaf-cutting ants is phenotypically plastic. Proc. R. Soc. B 274, 1625–1630 (doi:10.1098/rspb.2007.0347) 10.1098/rspb.2007.0347 PMC2169285 17301017

[RSPB20091415C17] Hughes W. O. H. Boomsma J. J. 2008 Genetic royal cheats in leaf-cutting ant societies. Proc. Natl Acad. Sci. USA 105, 5150–5153 (doi:10.1073/pnas.0710262105) 18339809 10.1073/pnas.0710262105 PMC2278208

[RSPB20091415C18] Hughes W. O. H. Eilenberg J. Boomsma J. J. 2002 Trade-offs in group living: transmission and disease resistance in leaf-cutting ants. Proc. R. Soc. Lond. B 269, 1811–1819 (doi:10.1098/rspb.2002.2113) 10.1098/rspb.2002.2113 PMC1691100 12350269

[RSPB20091415C19] Hughes W. O. H. Sumner S. Van Borm S. Boomsma J. J. 2003 Worker caste polymorphism has a genetic basis in *Acromyrmex* leaf-cutting ants. Proc. Natl Acad. Sci. USA 100, 9394–9397 (doi:10.1073/pnas.1633701100) 12878720 10.1073/pnas.1633701100 PMC170929

[RSPB20091415C20] Hughes W. O. H. Oldroyd B. P. Beekman M. Ratnieks F. L. W. 2008a Ancestral monogamy shows kin selection is key to the evolution of eusociality. Science 320, 1213–1216 (doi:10.1126/science.1156108) 18511689 10.1126/science.1156108

[RSPB20091415C21] Hughes W. O. H. Pagliarini R. Madsen H. B. Dijkstra M. J. Boomsma J. J. 2008b Antimicrobial defence shows an abrupt evolutionary transition in the fungus-growing ants. Evolution 62, 1252–1257 (doi:10.1111/j.1558-5646.2008.00347.x) 18266984 10.1111/j.1558-5646.2008.00347.x

[RSPB20091415C22] Jaccoud D. B. Hughes W. O. H. Jackson C. W. 1999 The epizootiology of a *Metarhizium* infection in mini-nests of the leaf-cutting ant *Atta sexdens rubropilosa*. Entomol. Exp. Appl. 93, 51–61 (doi:10.1023/A:1003830625680)

[RSPB20091415C23] Kermarrec A. Febvay G. Decharme M. 1986 Protection of leaf-cutting ants from biohazards: is there a future for microbiological control? In Fire ants and leaf-cutting ants: biology and management (eds Lofgren C. S. Vander Meer R. K.), pp. 338–355 Boulder, CO: Westview Press

[RSPB20091415C24] Kover P. X. Schaal B. A. 2002 Genetic variation for disease resistance and tolerance among *Arabidopsis thaliana* accessions. Proc. Natl Acad. Sci. USA 99, 11 270–11 274 (doi:10.1073/pnas.102288999) 10.1073/pnas.102288999 PMC123246 12172004

[RSPB20091415C25] Kraaijeveld A. R. Godfray H. C. J. 1997 Trade-off between parasitoid resistance and larval competitive ability in *Drosophila melanogaster*. Nature 389, 278–280 (doi:10.1038/38483) 9305840 10.1038/38483

[RSPB20091415C26] Little T. J. Ebert D. 1999 Associations between parasitism and host genotype in natural populations of *Daphnia* (Crustacea: Cladocera). J. Anim. Ecol. 68, 134–149 (doi:10.1046/j.1365-2656.1999.00271.x)

[RSPB20091415C27] Little T. J. Ebert D. 2000 The cause of parasitic infection in natural populations of *Daphnia* (Crustacea: Cladocera): the role of host genetics. Proc. R. Soc. Lond. B 267, 2037–2042 (doi:10.1098/rspb.2000.1246) 10.1098/rspb.2000.1246 PMC1690779 11416906

[RSPB20091415C28] Little T. J. Ebert D. 2001 Temporal patterns of genetic variation for resistance and infectivity in a *Daphnia*–microparasite system. Evolution 55, 1146–1152 11475050 10.1111/j.0014-3820.2001.tb00634.x

[RSPB20091415C29] Navajas M. Migeon A. Alaux C. Martin-Magniette M. L. Robinson G. E. Evans J. D. Cros-Arteil S. Crauser D. Le Conte Y. 2008 Differential gene expression of the honey bee *Apis mellifera* associated with *Varroa destructor* infection. BMC Genom. 9, 301 (doi:10.1186/1471-2164-9-301) 10.1186/1471-2164-9-301 PMC2447852 18578863

[RSPB20091415C30] Ortius-Lechner D. Gertsch P. J. Boomsma J. J. 2000a Variable microsatellite loci for the leaf cutter ant *Acromyrmex echinatior* and their applicability to related species. Mol. Ecol. 9, 114–116 (doi:10.1046/j.1365-294x.2000.00764-5.x) 10652084 10.1046/j.1365-294x.2000.00764-5.x

[RSPB20091415C31] Ortius-Lechner D. Maile R. Morgan E. D. Boomsma J. J. 2000b Metapleural gland secretion of the leaf-cutter ant *Acromyrmex octospinosus*: new compounds and their functional significance. J. Chem. Ecol. 26, 1667–1683 (doi:10.1023/A:1005543030518)

[RSPB20091415C32] Ortius-Lechner D. Maile R. Morgan E. D. Petersen H. C. Boomsma J. J. 2003 Lack of patriline-specific differences in chemical composition of the metapleural gland secretion in *Acromyrmex octospinosus*. Insectes Soc. 50, 113–119 (doi:10.1007/s00040-003-0640-1)

[RSPB20091415C33] Oster G. F. Wilson E. O. 1978 Caste and ecology in the social insects Princeton, NJ: Princeton University Press 740003

[RSPB20091415C34] Pigliucci M. 2005 Evolution of phenotypic plasticity: where are we going now? Trends Ecol. Evol. 20, 481–486 16701424 10.1016/j.tree.2005.06.001

[RSPB20091415C35] Poulsen M. Bot A. N. M. Currie C. R. Boomsma J. J. 2002a Mutualistic bacteria and a possible trade-off between alternative defence mechanisms in *Acromyrmex* leaf-cutting ants. Insectes Soc. 49, 15–19 (doi:10.1007/s00040-002-8271-5)

[RSPB20091415C36] Poulsen M. Bot A. N. M. Nielsen M. G. Boomsma J. J. 2002b Experimental evidence for the costs and hygienic significance of the antibiotic metapleural gland secretion in leaf-cutting ants. Behav. Ecol. Sociobiol. 52, 151–157 (doi:10.1007/s00265-002-0489-8)

[RSPB20091415C37] Poulsen M. Hughes W. O. H. Boomsma J. J. 2006 Differential resistance and the importance of antibiotic production in *Acromyrmex echinatior* leaf-cutting ant castes towards the entomopathogenic fungus *Aspergillus nomius*. Insectes Soc. 53, 349–355 (doi:10.1007/s00040-006-0880-y)

[RSPB20091415C38] Reeson A. F. Wilson K. Gunn A. Hails R. S. Goulson D. 1998 Baculovirus resistance in the noctuid *Spodoptera exempta* is phenotypically plastic and responds to population density. Proc. R. Soc. Lond. B 265, 1787–1791 (doi:10.1098/rspb.1998.0503)

[RSPB20091415C39] Rolff J. Siva-Jothy M. T. 2003 Invertebrate ecological immunology. Science 301, 472–475 (doi:10.1126/science.1080623) 12881560 10.1126/science.1080623

[RSPB20091415C40] Spivak M. Reuter G. S. 2001 *Varroa destructor* infestation in untreated honey bee (Hymenoptera: Apidae) colonies selected for hygienic behavior. J. Econ. Entomol. 94, 326–331 11332821 10.1603/0022-0493-94.2.326

[RSPB20091415C41] Sumner S. Hughes W. O. H. Boomsma J. J. 2003 Evidence for differential selection and potential adaptive evolution in the worker caste of an inquiline social parasite. Behav. Ecol. Sociobiol. 54, 256–263 (doi:10.1007/s00265-003-0633-0)

[RSPB20091415C42] Sumner S. Hughes W. O. H. Pedersen J. S. Boomsma J. J. 2004 Ant parasite queens revert to mating singly. Nature 428, 35–36 (doi:10.1038/428035a) 14999273 10.1038/428035a

[RSPB20091415C43] Vijendravarma R. K. Kraaijeveld A. R. Godfray H. C. J. 2009 Experimental evolution shows *Drosophila melanogaster* resistance to a microsporidian pathogen has fitness costs. Evolution 63, 104–114 (doi:10.1111/j.1558-5646.2008.00516.x) 18786186 10.1111/j.1558-5646.2008.00516.x

[RSPB20091415C44] Villesen P. Murakami T. Schultz T. R. Boomsma J. J. 2002 Identifying the transition between single and multiple mating of queens in fungus-growing ants. Proc. R. Soc. Lond. B 269, 1541–1548 (doi:10.1098/rspb.2002.2044) 10.1098/rspb.2002.2044 PMC1691065 12184823

[RSPB20091415C45] Weber N. A. 1972 Gardening ants: the attines. Mem. Am. Phil. Soc. 92, 1–146

[RSPB20091415C46] Wetterer J. K. 1999 The ecology and evolution of worker size-distribution in leaf-cutting ants (Hymenoptera: Formicidae). Sociobiology 34, 119–144

[RSPB20091415C47] Wilson E. O. 1980 Caste and division of labor in leaf-cutter ants (Hymenoptera: Formicidae: *Atta*). I. The overall pattern in *A. sexdens*. Behav. Ecol. Sociobiol. 7, 143–156 (doi:10.1007/BF00299520)

[RSPB20091415C48] Wilson K. Thomas M. B. Blanford S. Doggett M. Simpson S. J. Moore S. L. 2002 Coping with crowds: density-dependent disease resistance in desert locusts. Proc. Natl Acad. Sci. USA 99, 5471–5475 (doi:10.1073/pnas.082461999) 11960003 10.1073/pnas.082461999 PMC122793

